# Burden of Road Traffic Injuries in Tanzania: One-Year Prospective Study of Consecutive Patients in 13 Multilevel Health Facilities

**DOI:** 10.1155/2021/4272781

**Published:** 2021-11-10

**Authors:** Hendry R. Sawe, Sveta Milusheva, Kevin Croke, Saahil Karpe, Meyhar Mohammed, Juma A. Mfinanga

**Affiliations:** ^1^Department of Emergency Medicine, Muhimbili University of Health and Allied Sciences, Dar es Salaam, Tanzania; ^2^Department of Emergency Medicine, Muhimbili National Hospital, Dar es Salaam, Tanzania; ^3^Development Impact Evaluation Group, World Bank, Washington, DC, USA; ^4^Harvard T. H. Chan School of Public Health, Boston, MA, USA; ^5^Lyft, San Francisco, CA, USA

## Abstract

**Background:**

Road traffic injuries (RTIs) pose a severe public health crisis in Sub-Saharan Africa (SSA) and specifically in Tanzania, where the mortality due to RTIs is nearly double the global rate. There is a paucity of RTI data in Tanzania to inform evidence-based interventions to reduce the incidence and improve care outcomes. A trauma registry was implemented at 13 health facilities of diverse administrative levels in Tanzania. In this study, we characterize the burden of RTIs seen at these health facilities.

**Methods:**

This was a one-year prospective descriptive study utilizing trauma registry data from 13 multilevel health facilities in Tanzania from 1 October 2019 to 30 September 2020. We provide descriptive statistics on patient demographics; location; share of injury; nature, type, and circumstances of RTI; injury severity; disposition; and outcomes.

**Results:**

Among 18,553 trauma patients seen in 13 health facilities, 7,416 (40%) had RTIs. The overall median age was 28 years (IQR 22–38 years), and 79.3% were male. Most road traffic crashes (RTC) occurred in urban settings (68.7%), involving motorcycles (68.3%). Motorcyclists (32.9%) were the most affected road users; only 37% of motorcyclists wore helmets at the time of the crash. The majority (88.2%) of patients arrived directly from the site, and 49.0% used motorized (two- or three-) wheelers to travel to the health facility. Patients were more likely to be admitted to the ward, taken to operating theatre, died at emergency unit (EU), or referred versus being discharged if they had intracranial injuries (27.8% vs. 3.7%; *p* < 0.0001), fracture of the lower leg (18.9% vs. 6.4%; *p* < 0.0001), or femur fracture (12.9% vs. 0.4%; *p* < 0.0001). Overall, 36.1% of patients were admitted, 10.6% transferred to other facilities, and mortality was 2%.

**Conclusions:**

RTCs are the main cause of trauma in this setting, affecting mostly working-age males. These RTCs result in severe injuries requiring hospital admission or referral for almost half of the victims. Motorcyclists are the most affected group, in alignment with prior studies. These findings demonstrate the burden of RTCs as a public health concern in Tanzania and the need for targeted interventions with a focus on motorcyclists.

## 1. Background

Road traffic injuries (RTIs) contribute significantly to the global burden of diseases posing a particularly severe public health crisis in Sub-Saharan Africa (SSA) and specifically in Tanzania, where the mortality due to RTIs is nearly double the global rate [1]. Unfortunately, the latest multicountry survey reveals that very few countries in the SSA region have developed systematic emergency medical services (EMS) and prehospital care systems at scale; this has the potential to affect the outcomes for road crash victims [2].

In Tanzania, RTIs contribute significantly to the burden of disease, with significant morbidity and mortality among accident victims [3–5]. Like most low- and middle-income countries (LMICs), Tanzania lacks formal trauma care systems that further contribute to its challenges in addressing the large burden of injuries [6]. Furthermore, recent studies have suggested that healthcare facilities in this context are not adequately equipped to meet trauma care needs and face major gaps in coordinated emergency response [7]. Despite the high rates of RTI-related deaths in Tanzania [8], there is a gap in detailed trauma data that could inform policymakers about factors in emergency care that affects RTIs. This gap provides an opportunity to improve postinjury care and outcomes for RTI patients, through the implementation of EMS that incorporates reliable trauma and crash data. One of the key impediments identified for the development of robust EMS systems is the lack of data-driven information management systems that record longitudinal patient-level trauma data to provide region-specific evidence to improve outcomes on postcrash response [9]. In an effort to improve postcrash care and reduce fatalities from RTIs, the Government of Tanzania supported by the World Bank planned to implement a pilot EMS on the busy A7 highway connecting the North-South corridor of Tanzania [10]. In order to understand the health impacts of this pilot EMS in Tanzania, we implemented a multisite prospective trauma registry (TR) data collection at emergency units (EUs) of 13 multilevel health facilities that include the diverse scale of the administrative structure of Tanzania's public health infrastructure. In this manuscript, we characterize the burden of RTIs in these facilities, as a crucial step of setting a baseline for the future impact evaluation of EMS implementation in Tanzania and other similar settings in LMICs.

## 2. Methods

### 2.1. Study Design and Population

This was a one-year prospective descriptive study of all RTI patients presenting to 13 multilevel health facilities in Tanzania from 1 October 2019 to 30 September 2020.

### 2.2. Study Setting

The United Republic of Tanzania is a lower-middle-income country with a population of 60 million people [11, 12]. The public health system is organized in a pyramidal structure from the lowest level of the primary dispensary, followed by the health center, district hospital, regional hospital, national, and consultant hospitals [13]. There is no formal trauma care system and no formal prehospital system.

In an effort to mitigate the burden of RTIs, the Government of Tanzania, supported by the World Bank, planned a pilot implementation of EMS along the A7 highway that connects the north and south of Tanzania. This pilot EMS implementation included the creation of an ambulance dispatch center, activation of an emergency access telephone number, training community first responders, paramedics, fire safety personnel and drivers, procuring and equipping ambulances, and renovation of emergency units (EUs) in 6 health facilities located within 2 km of the A7 highway (from Dar es Salaam to Morogoro) that included 2 regional hospitals (Tumbi and Morogoro), 3 health centers (Kimara, Chalinze, and Mikumi), and 1 dispensary (Fulwe).

**Figure 1:**
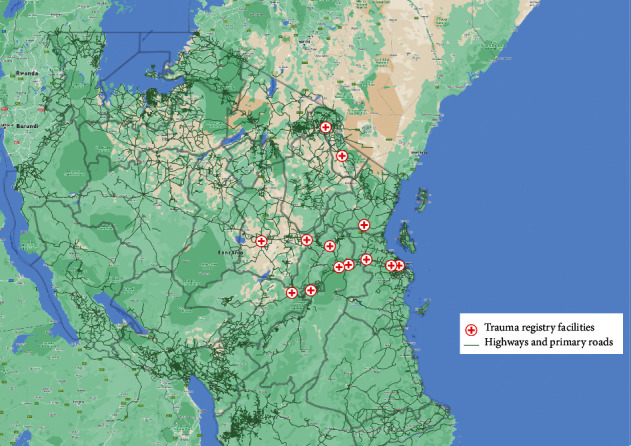
Map of Tanzania showing the location of each health facility.

In order to understand the impact of this pilot EMS implementation, we set up a trauma registry to enroll all injured patients at EUs of 13 public health facilities (Figure 1) that include all 6 health facilities involved in the pilot EMS implementation and 7 additional (comparison group) not part of the EMS implementation. The 7 additional (comparison group) facilities included 2 regional hospitals (Dodoma and Mawenzi), 3 district hospitals (Same, Korogwe, and Mvomero), and 2 health centers (Mkata and Gairo), all of which were located on a different though comparable highway.

### 2.3. Data Source

In EUs of each of the 13 health facilities, we implemented a paper-based standardized trauma form that had previously been developed and piloted at 5 different regional hospitals in Tanzania [14], prior to the launch of this project. This standardized trauma form was adopted and modified from the World Health Organization (WHO) standardized trauma form [15]. Prior to this implementation, we further modified and repiloted the form to ensure additional RTI variables could be collected. The trauma form was used for clinical documentation, and it had a carbonless copy to allow the duplication of information to be used for abstracting data into a digital platform hosted through an online data capture software Research Electronic Data Capture (REDCap; © REDCap version 7.2.2, Vanderbilt, Nashville, TN, USA).

In each health facility, injured patients presenting after an RTC were manually recorded into the trauma forms by clinicians and the research assistant (RA). The data from manual trauma forms were entered into the online REDCap system by the RA once the care process had been completed. The principal investigators received copies of the completed trauma forms from each of the sites for verification and data quality validation.

### 2.4. Personnel Training

In each of the 13 health facilities, we recruited and trained a trauma data coordinator (TDC) and RA who supported the data collection process. TDCs were healthcare providers (clinician or nurse) in the facility, which provided site project oversight and ensured buy-in and compliance. Both TDCs and RAs received dedicated training that focused on an overview of primary trauma care, the context of impact evaluation, and the use of the trauma registry paper form and the REDCap tool using digital tablets.

### 2.5. Data Analysis

Data from REDCap was exported into the Stata 16 StataCorp, College Station, TX, USA, for analysis. Descriptive statistics are presented as mean and median with corresponding standard deviation and interquartile range as appropriate. The final EU diagnoses were coded using the International Classification of Diseases (ICD) 10, and a chi-square test was used to test the categorical variables. The map of the location of facilities was constructed using a shapefile of the facilities in the QGIS Development Team (2021) Geographic Information System. In order to understand the share of RTC at a district level, we created a heat map using R Studio 2020 (Boston, MA).

## 3. Results

### 3.1. Patient Characteristics

A total of 18,553 trauma patients were seen in all health facilities, out of which 7,416 (40%) had RTIs. Among those who had RTIs, 5,862 (79.3%) were male, and the overall median age was 28 years (interquartile range 22–38 years). Petty traders 1,790 (25.3%), drivers 1,495 (21.1%), farmers 1,322 (18.7%), and students 806 (11.4%) were the most common occupations recorded. Most patients 6,455 (88.2%) presented directly from the scene of the crash, and 3,632 (49%) used a motorized (two- or three-) wheeler as a mode of arrival to the facility. Only 497 (6.7%) used ambulances to arrive at the facility. In the EU, 94% of patients were triaged as priority or emergency cases requiring immediate emergency care (Table 1).

### 3.2. Description of Nature of RTI with Risk Factors for Serious Injuries

Most RTC victims (65.7%) came from urban settings, as compared to (60.5%) of all trauma victims broadly from urban settings. Additionally, of victims who were in a motorized vehicle, over two-thirds were the drivers or passengers of motorcycles (67.9%). A majority of motorcycle drivers and passengers (62.6%) were not wearing helmets at the time of the crash, and similarly, among the vehicle occupants, only 17.4% had seat belt protection at the time of the accident. Motorcyclists and bicyclists accounted for over half (53%) of the patients involved in RTCs and nonmotorized users (pedestrians and cyclists) accounted for 42.2% of the patients. Almost half of these nonmotorized users (48%) were hit by a motorcycle. Alcohol use was missing or reported unknown in 69.4% of injured patients; alcohol use was reported in 1.2% of cases. Morning (0600–1159 hours) and evening (1800–2359 hours) rush hours accounted for most of the accidents at 35.6% and 26.2%, respectively. A plurality of patients 1781 (29.8%) arrived in the EU 1–2 hours after the crash, while only 750 (12.5%) arrived in less than 30 minutes (Table 2).

### 3.3. ICD-10 Diagnosis and Final EU Disposition

Multiple superficial injuries (35.6%) and open wounds (26.6%) were leading diagnoses, and patients with these diagnoses were more likely to be discharged. Overall, a majority of patients with intracranial injury 977/1110 (88.0%), femur fracture 453/466 (97.2%), and fracture of the lumbar spine and pelvis 41/44 (93.2%) were admitted to the ward, taken to the operating theatre, died at EU, or referred to other facilities. Patients were more likely to be admitted to the ward, taken to operating theatre, died at EU, or referred to other facilities versus being discharged if they had intracranial injuries (27.8% vs. 3.7%; *p* < 0.0001), fracture of the lower leg (18.9% vs. 6.4%; *p* < 0.0001), or femur fracture (12.9% vs. 0.4%; *p* < 0.0001; Table 3).

### 3.4. Final EU Disposition by Patient's Role

Overall, 3540 (47%) patients were either admitted or referred to higher-level health facilities with more resources and personnel. Pedestrians have the lowest rate of being discharged home 669 (42.2%), signaling a higher severity of injuries suffered, while 1,214 (51.2%) of motorcyclists were discharged home from the EU. Overall, 144 (1.9%) of RTC victims died (including 73 (0.98%) deaths in the EU and 71 (0.96%) in transit or on the scene of the crash). Among those who died, the pedestrian 31/144 (21.5%) accounted for the highest proportion, followed by cyclists, which accounted for 30/144 (20.8%; Table 4).

### 3.5. Road Traffic Crash Share by District

**Figure 2:**
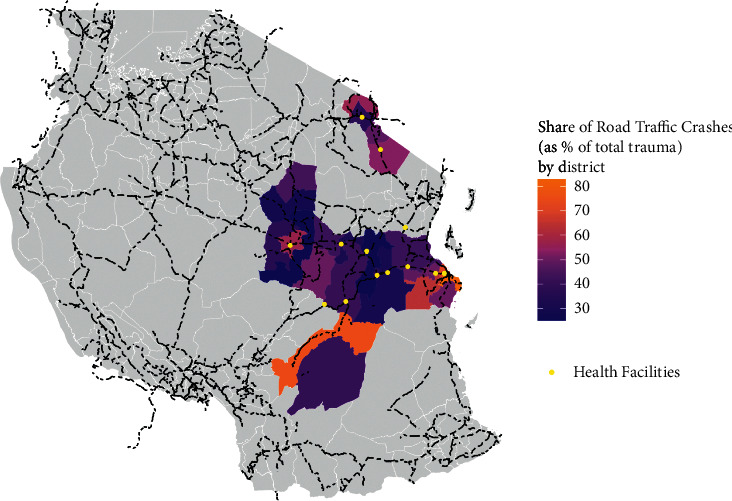
Heat map of accident hotspots^*∗*^. Note: This map shows road traffic crashes as a percentage share of total trauma in the trauma registry at the district level. The colored region represents 30 districts surrounding the facilities (seen in yellow dots), which were recorded in the trauma registry. Lighter shade represents a higher share of road traffic crashes recorded in a given district. The gray portion refers to other districts in Tanzania, which were not recorded in the trauma registry. The dashed black lines represent the highways and other primary roads in Tanzania.

There is geographic variation in the burden of road traffic crashes across the districts surrounding the health facilities. We found that Temeke (81%), Kigamboni (77%), Ilala (82%), Kilombero (75%), Kinondoni (70%), Siha (57%), Dodoma (57%), Rombo (54%), Same (53.08%), Kibaha (50%), and Kisarawe (63%) have more than half of their recorded trauma as RTC (Figure 2). It should be noted that for some of these districts, the denominator (total number of trauma cases) could be small, especially those districts further away from health facilities in which data was recorded.

## 4. Discussion

In this prospective registry-based study of 13 multilevel health facilities in Tanzania, we found a substantial burden of injuries resulting from RTCs, with the majority of patients having injuries serious enough to require admission or transfer to a higher level of trauma care. This multisite implementation of a trauma registry and diverse nature of health facilities ranging from dispensary to regional level hospital provides a broader picture of the burden of RTIs received in health facilities, compared to previously published literature.

Similar to previously published literature in LMICs [3, 5, 16], motorcyclists accounted for the largest proportion of victims of injuries presenting to these facilities, with the majority of vehicles involved in crashes being a motorcycle. We found a very low threshold of wearing protective equipment (helmets and seat belts) for safety among all vehicle occupants at the time of the crash. Given the amount of evidence supporting the impact of protective equipment in RTC [17], these findings provide a great opportunity for targeted road safety policy and regulation interventions that have a substantial and instant impact on the outcome of injuries resulting from RTCs [18].

Bicyclists and pedestrians each make up around 20% of road crash victims, which is in line with other LMIC studies [19]. Not surprisingly, a higher share of pedestrians experience potentially more severe trauma as compared to other road users. Limitations on road infrastructure such as lack of dedicated walk and bicycle lanes, as well as failure to observe road safety regulations, might be attributed to this high incidence. Improvement in road infrastructure and instituting appropriate legal guidelines has been shown to reduce the incidence of RTCs in most high-income countries (HICs) [20]; hence, similar interventions in Tanzania will likely have an impact on RTCs in these groups.

Interestingly, we found a peak of RTC in the day and evening time that corresponds to the peak traffic times in Tanzania, and the majority of cases occurring at night were severe as compared to daytime cases. Improvement of road infrastructure and targeted road surveillance by law enforcing agencies during peak hours to enforce safe driving is likely to have an impact in reducing the incidence of RTCs in these settings. Rapid urbanization and motorization have been associated with increased incidents of RTC in most LMICs [21]; likewise, in our study, over two-thirds of RTCs were reported to have occurred in urban settings. Over half of victims coming to these facilities were aged between 15 and 34 years of age, with the majority being male, working as petty traders or professional drivers, findings in concurrence with global data on RTC [1].

Most patients presented directly from the scene of the crash, with almost half of them using motorized (two- or three-wheelers) as means of transport to the facilities. Formal emergency transports such as ambulances were rarely used even in serious cases. Consequently, most patients arrived at the health facilities more than one hour after the injury incidents, which is high compared to what has been observed in other studies in some similar settings and in HICs [22]. As shown in previous studies, lack of formal prehospital care is one of the major challenges in improving outcomes of immediate postcrash care [23–25] and might have an impact on both morbidity and mortality.

In emergency care settings, the initial triage score of injury victims is known to be associated with injury severity as well as a clinical outcome [26]. Over 90% of patients were triaged at priority or emergency triage level, requiring immediate life-saving interventions in the EUs to save lives and/or reduce the likelihood of developing lifelong disabilities. These findings suggest the future need to assess the association of triage scale with severity or clinical outcomes and to further evaluate, design, and provide dedicated training on triage processes to ensure proper prioritization of care and patient transfer.

Similar to previously published studies [27, 28], head injuries accounted for the largest category of serious injuries highlighting the resource-intensive nature of these injuries. In Tanzania, there is a scarcity of advanced neurosurgical care, with only one tertiary-level trauma care center that is fully equipped with resources to provide advanced neurosurgical care to these patients [29]. We believe a study of long-term outcomes among these patients will help quantify the actual burden and impact of head injuries to these patients and help develop a clear protocol for admission, discharge, and referral to optimize outcomes.

### 4.1. Limitations

This study was conducted on a purposefully selected sample of health facilities located within 2 km of busy highways in Tanzania and may not necessarily reflect the actual burden of RTC across other regions. However, the diverse nature of these health facilities (ranging from dispensary level to regional hospital level) provided an opportunity to understand the burden at different levels of care across the country. The data collection was also affected by COVID-19 in different ways. First, the partial lockdown in the early months of COVID-19 in Tanzania might have led to a decline in the number of accidents. Nevertheless, the partial lockdown in Tanzania was only for a brief period of time (approximately 10 weeks), and we do not find a sudden drop in RTI cases, instead of finding consistency in the number of cases across months. Second, the COVID-19 pandemic led to a withdrawal of RAs from the sites of data collection to reduce their risk of being exposed; this affected the quality and quantity of data, as clinicians were not able to enter all the data without the assistance of the RAs. Third, due to the COVID-19 pandemic, there was a deviation of cases that were dead on arrival to the mortuary without passing through the EUs, and this practice affected the observations for death at the crash scene or in transit to EUs, and it might have resulted in underestimates of the total deaths.

## 5. Conclusion

RTCs are the main cause of trauma in Tanzania affecting mostly male, economically productive age groups, and they result in severe injuries in almost half of victims. Motorcyclists remain the most commonly affected group; in alignment with several prior studies, however, pedestrians and cyclists also constituted a substantial proportion. The impact of lack of formal prehospital care was demonstrated by utilization of private means of transport and delays in RTIs patients' arrival at the health facility. These findings demonstrate the burden of RTCs as a first-order public health concern in Tanzania and signify the need for targeted policy efforts towards reducing RTCs and improving postinjury care at all levels.

## Figures and Tables

**Table 1:** RTI patient characteristics.

	*N* = 7,416
Sex^ň^	*n* (%)
Male	5,862 (79.3%)
Female	1,529 (20.7%)

Age	
Median (IQR) years	28 years (IQR: 22–38 years)

Age groups	*n* (%)
<5 years	146 (1.99)
5–14 years	482 (6.5)
15–24 years	1,922 (26.14)
25–34 years	2,426 (32.9)
35–44 years	1,154 (15.6)
45–54 years	658 (8.9)
55–64 years	308 (4.2)
>65 years	258 (3.5)

Triage level^*∗*^	
Emergency	1,736 (23.4)
Priority	5,234 (70.6)
Queue	330 (4.5)

Referral status	
Direct from crash site	6,455 (88.2)
Referred	867 (11.8)

Occupation	
Petty trader	1,790 (25.3)
Driver	1,495 (21.1)
Farmer	1,322 (18.7)
Student	806 (11.4)
Manual laborer	221 (3.0)
Others^*ρ*^	1,451(20.0)

Mode of arrival^*Ħ*^	
Motorcycle	2,347 (31.7)
Car	1,806 (24.5)
Tricycle	1,285 (17.3)
Minibus	781 (10.5)
Ambulance	497 (6.7)
Walk-in	323 (4.4)
Bus	10 (0.13)
Bicycle	16 (0.2)
Others	293 (3.95)

^*∗*^116 (1.6%) observations are missing triage status, ^**ň**^25 (0.3%) are missing gender, and ^Ħ^56 (0.8%) missing mode of arrival to EU. ^*p*^includes office worker, military, mining, craftsman, health worker, unemployed, retired individuals, housewife, unknown, and other unspecified.

**Table 2:** Description of nature of RTI with risk factors for serious injuries.

	Overall	Nondischarged^*∗*^	Percentage of nondischarged^⦽^
	*N* (%)	*n* (%)	*n*/*N* (%)
Vehicle involved in RTC^*ρ*^	*N* = 3, 480	*n* = 1, 647	
Motorcycle	2, 375 (67.91)	1, 151 (69.62)	1, 151/2, 375 (48.46)
Bus/minibus	533 (15.24)	222 (13.43)	222/533 (41.65)
Car	536 (15.33)	257 (15.55)	257/536 (47.94)
Others	36 (1.02)	17 (1.02)	17/36 (47.22)

Patient's role on the road^*μ*^	*N* = 7226	*n* = 3550	*n*/*N* (%)
Motorcyclist	2, 375 (32.92)	1, 151 (31.86)	1, 151/2, 375 (48.46)
Bicyclist	1, 454 (20.12)	709 (19.62)	709/1, 454 (48.76)
Pedestrian	1, 590 (22.04)	901 (25.94)	901/1, 590 (56.67)
Bus/minibus occupant	533 (7.38)	222 (6.14)	222/533 (41.65)
Car occupant	536 (7.43)	271 (7.11)	271/536 (50.56)
Others	738 (10.23)	301 (8.33)	301/738 (40.79)

RTC site^ň^	*N* = 7092	*n* = 3411	*n*/*N* (%)
Urban site	4, 873 (65.71)	2, 372 (65.65)	2, 372/4, 873 (48.68)
Rural site	2, 167 (29.22)	1, 009 (27.93)	1, 009/2, 167 (46.56)
Unknown	52 (0.73)	30 (0.88)	30/52 (57.69)

Use of safety equipment^*∗∗*^			
Seat belt	96/553 (17.36)	46/266 (17.29)	46/96 (47.92)
Helmet	889/2, 375 (37.43)	410/1, 151 (35.62)	410/889 (46.12)

Alcohol status^Ħ^	*N* = 4, 013	*n* = 2, 106	*n*/*N* (%)
None reported	1, 182 (29.45)	524 (24.88)	524/1, 182 (44.33)
Confirmed use	47 (1.17)	30 (1.42)	30/47 (63.82)
Unknown or missing	2, 784 (69.37)	1, 552 (73.69)	1, 552/2, 784 (55.75)

Time of accident	*N* = 7251	*n* = 3555	*n*/*N* (%)
0000–0559 hrs	651 (8.78)	340 (9.41)	340/651 (52.22)
0600–1159 hrs	2, 639 (35.59)	1, 175 (32.53)	1, 175/2, 639 (44.52)
1200–1759 hrs	2, 017 (27.20)	984 (27.23)	984/2, 017 (48.79)
1800–2359 hrs	1, 944 (26.21)	1, 056 (29.23)	1, 056/1, 944 (54.32)

Duration of arrival^*φ*^	*N* = 5, 985	*n* = 2, 762	*n*/*N* (%)
<30 minutes	750 (12.53)	298 (10.79)	298/750 (39.73)
30–60 minutes	1, 787 (29.86)	804 (29.11)	804/1, 787 (44.99)
60–120 minutes	1, 781 (29.76)	822 (29.76)	822/1, 781 (46.15)
120–180 minutes	773 (12.92)	400 (14.48)	400/773 (51.75)
>180 minutes	894 (14.94)	438 (15.86)	438/894 (48.99)

^*∗*^Those who required hospital admission, required emergency operation, or died after injury. ^***ρ***^Include patients with a role on the road as driver or passenger. ^Ħ^ Includes only driver, cyclist, and pedestrian. ^*∗∗*^Applicable for driver and passenger of car and motorcycle. ^***μ***^Details of role on the road missing in 190 patients and 63 missing hospital dispositions. ^**ň**^ Crash site missing in 324 cases and 203 missing hospital disposition. ^ự^Time to accident was missing in 165 cases. ^*φ*^Duration of arrival that excludes referred cases.

**Table 3:** ICD-10 diagnosis and final EU disposition.

All patients^ň^	Discharged	Nondischarged^*μ*^	Proportion of nondischarged	*p* value^ự^
ICD-10 diagnosis^*∗*^	*N* = 7, 128	3, 613	3, 515		*n*/*N* (%)
Multiple superficial injuries, unspecified	2, 535 (35.6)	1, 698 (47.0)	837 (23.8)	837/2, 535 (33.0)	*p* < 0.0001

Open wound of unspecified body region	1, 899 (26.6)	1, 354 (37.5)	545 (15.5)	545/1, 899 (28.7)	*p* < 0.0001

Intracranial injury	1, 110 (15.6)	133 (3.7)	977 (27.8)	977/1, 110 (88.0)	*p* < 0.0001

Fracture of lower leg, including ankle	898 (12.6)	233 (6.4)	665 (18.9)	665/898 (74.1)	*p* < 0.0001

Fracture of femur	466 (6.5)	13 (0.4)	453(12.9)	453/466 (97.2)	*p* < 0.0001

Fracture of forearm	251 (3.5)	94 (2.6)	157 (4.5)	157/251 (62.5)	*p* < 0.0001

Fracture of shoulder and upper arm	230 (3.2)	62 (1.7)	168 (4.8)	168/230 (73.0)	*p* < 0.0001

Unspecified injury of thorax	170 (2.4)	39 (1.1)	131 (3.7)	131/170 (77.1)	*p* < 0.0001

Sprain and strain of other and unspecified parts of foot	115 (1.6)	95 (2.6)	20 (0.6)	20/115 (17.4)	*p* < 0.0001

Fracture of unspecified body region	104 (1.5)	17 (0.5)	87 (2.5)	87/104 (83.7)	*p* < 0.0001

Fracture of skull and facial bones	84 (1.2)	19 (0.5)	65 (1.8)	65/84 (77.4)	*p* < 0.0001

Dislocation, sprain, and strain of joints and ligaments of shoulder girdle	79 (1.1)	36 (1.0)	43 (1.2)	43/79 (54.4)	*p*=0.4180

Dislocation, sprain, and strain of joints and ligaments of knee	79 (1.1)	21 (0.6)	58 (1.7)	58/79 (73.4)	*p* < 0.0001

Fracture at wrist and hand level	79 (1.1)	41 (1.1)	38 (1.1)	38/79 (48.1)	*p*=1.0

Fracture of lower leg, including ankle	64 (0.9)	20 (0.6)	44 (1.3)	44/64 (68.8)	*p*=0.0023

Open wounds of head, neck, and trunk	62 (0.9)	32 (0.9)	30 (0.9)	30/62 (48.4)	*p*=1.0

Dislocation, unspecified	58 (0.8)	17 (0.5)	41 (1.2)	41/58 (70.7)	*p*=0.0012

Injury, unspecified	49 (0.7)	25 (0.7)	24 (0.7)	24/49 (48.9)	*p*=1.0

Fracture of lumbar spine and pelvis	44 (0.6)	3 (0.1)	41 (1.2)	41/44 (93.2)	*p* < 0.0001

Dislocation, sprain, and strain of joints and ligaments at ankle and foot level	44 (0.6)	18 (0.5)	26 (0.7)	26/44 (59.1)	*p*=0.2738

^*∗*^One patient may have more than one diagnosis, and they may appear more than once. ^**ň**^Overall 43 patients had no final EU disposition, and 245 were missing final EU diagnosis. ^***μ***^Nondischarged patients included patients who were admitted to the ward, taken to the operating theatre, died at EU, or referred to other facilities. ^ự^*P* value for the difference of discharged versus nondischarged.

**Table 4:** Final EU disposition by patient's role.

Disposition	Overall *n* = 7416	Pedestrian *n* = 1590	Motorcyclist *n* = 2375	Cyclist *n* = 1454	Car occupant *n* = 536
	*n* (%)	*n* (%)	*n* (%)	*n* (%)	*n* (%)
Admitted to hospital^*∗*^	2,675 (36.07)	766 (48.20)	859 (36.25)	477 (32.97)	163 (30.60)
Discharged home	3,708 (50.00)	669 (42.16)	1,214 (51.22)	731 (50.52)	259 (48.87)
OT admission	80 (1.08)	18 (1.13)	31 (1.31)	11 (0.76)	6 (1.13%)
Referred	785 (10.59)	105 (6.60)	243 (10.25)	202 (13.96)	78 (14.72)
Died^**ň**^	144 (1.94)	31 (1.95)	26 (1.09)	30 (2.06)	27 (5.03)
Unknown	24 (1.28%)	1 (0.06)	2 (0.42)	3 (0.96)	3 (3.73)

^*∗*^Include wards and ICU admission. ^**ň**^Include 73 (0.98%) deaths in the EU and 71 (0.96%) in transit or on the scene of the crash.

## Data Availability

The data sets used and/or analyzed during the current study are available on request from principal administrators.
